# Hypercoagulable State in COVID-19: A Case Series of Three Patients

**DOI:** 10.7759/cureus.8872

**Published:** 2020-06-27

**Authors:** Gideon Logan, Larissa Dub, Emily Drone, Latha Ganti, Amanda L Webb

**Affiliations:** 1 Emergency Medicine, University of Central Florida College of Medicine, Orlando, USA; 2 Emergency Medicine, University of Central Florida/Hospital Corporation of America/Graduate Medical Education Consortium and Osceola Regional Medical Center, Olrando, USA; 3 Emergency Medicine, Envision Physician Services, Nashville, USA; 4 Emergency Medicine, University of Central Florida College of Medicine/Hospital Corporation of America Graduate Medical Education Consortium of Greater Orlando, Orlando, USA; 5 Emergency Medical Services, Polk County Fire Rescue, Bartow, USA

**Keywords:** covid-19, sars-cov-2, novel coronavirus, hypercoagulable, prothrombotic, acute pulmonary embolism, dvt, venous thromboembolsim

## Abstract

The novel coronavirus disease 2019 (COVID-19) pandemic, caused by severe acute respiratory syndrome coronavirus 2 (SARS-CoV-2) that originated in China in late 2019, has caused significant morbidity and mortality worldwide. Although fever, cough, and shortness of breath have been recognized as hallmark symptoms, other lesser known complications continue to be described. We report a series of three patients who presented to the emergency department, who tested positive for COVID-19, and were found to have or subsequently developed thromboembolic complications.

## Introduction

Coronavirus or COVID-19 is a novel disease that originated in China in December 2019. There are 213 countries, areas, or territories where COVID-19 has been reported by the WHO. Since origination, according to the WHO on April 14, 2020, there have been 1,844,863 confirmed cases resulting in 117,021 deaths worldwide [1]. Common symptoms include fever, cough, shortness of breath, and body aches. Headache, diarrhea, and sore throat have also been reported although less frequently. Other less reported complications include anosmia, ageusia, and hypercoagulability [2]. We report three cases of COVID-19 infection resulting in thromboembolic complications.

## Case presentation

Case 1

**Figure 1:**
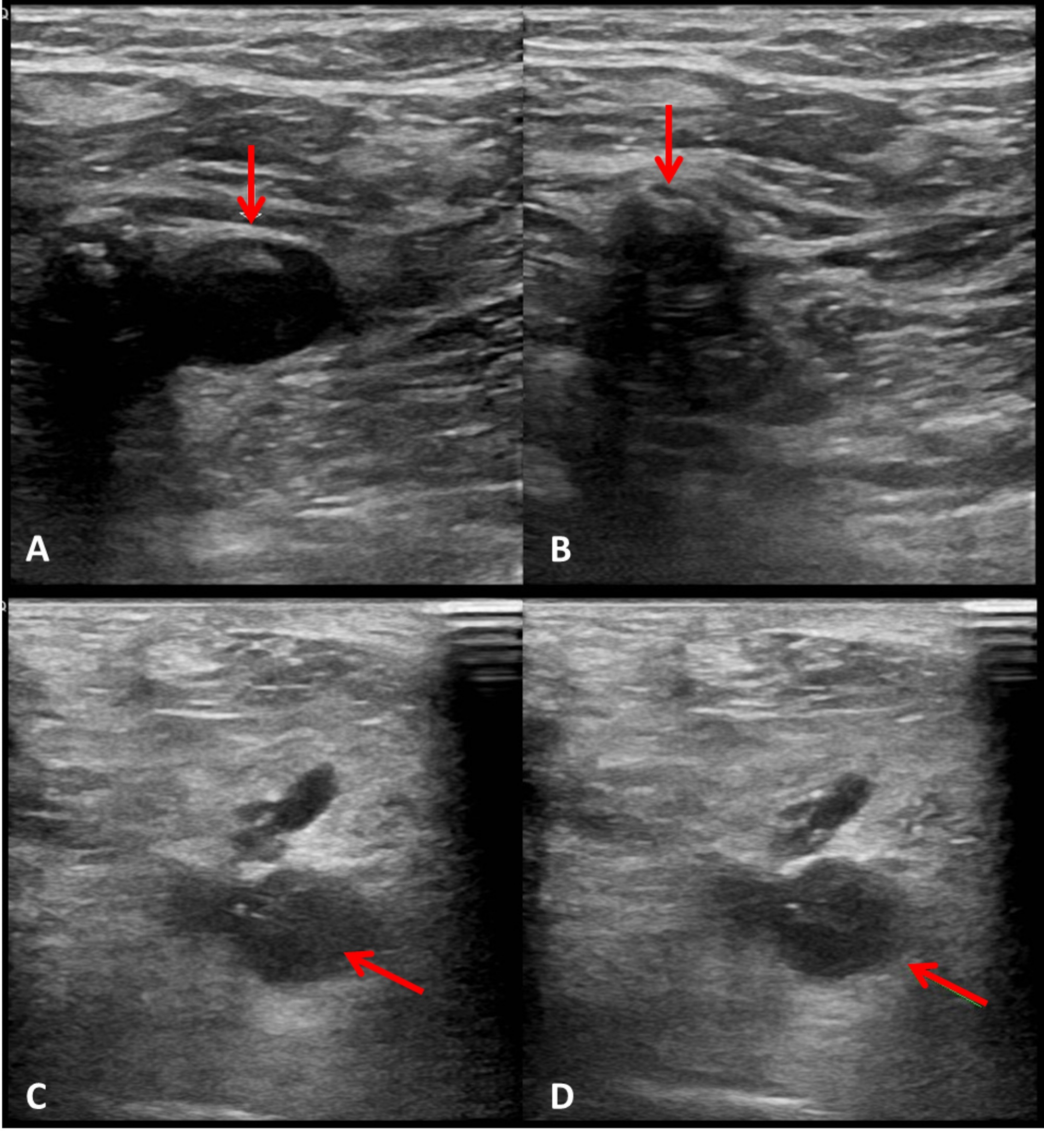
Ultrasonography of the right leg. (A) Common femoral vein (red arrow) without compression and (B) with compression. The common femoral vein is incompressible due to the thrombus. (C) Popliteal vein (red arrow) and arteries without compression, (D) and incompressible popliteal vein with thrombus (red arrow).

A 64-year-old female with a past medical history of atrial fibrillation, chronic obstructive pulmonary disease (COPD), diabetes, hypertension, hyperlipidemia, and one pack per day smoking presented to the emergency room for evaluation of right leg pain. She had been experiencing right leg pain for approximately three to four days, but denied redness, swelling, or known injury. She underwent left knee arthroscopy approximately three to four months prior and denied any recent long distance travel, history of malignancy, or history of deep vein thrombosis (DVT) or pulmonary embolism (PE). She denied any recent exposure to a COVID-positive patient and had been confined to her home for the last several weeks. The patient scheduled an appointment with her primary care doctor the day of presentation regarding her leg pain. A duplex ultrasound was performed which demonstrated an occlusive thrombus in the right popliteal vein and posterior tibial veins as well as a nonocclusive thrombus in the right common femoral vein (Figure 1). She was sent to the emergency room for further evaluation.

In triage, the patient endorsed a mild increase in her baseline dyspnea, but denied chest pain or a new cough. Her triage vitals were significant for a temperature of 36.9°C, a respiratory rate of 28 breaths per minute, a heart rate of 128 beats per minute, a blood pressure of 135/81 mmHg, and an oxygen saturation of 72% on room air. Her electrocardiogram (EKG) showed atrial fibrillation with rapid ventricular response at a rate of 150 beats per minute, left axis deviation, QTc 401 ms, and no ST-segment elevations or depressions.

Upon evaluation, the patient was alert and oriented. Her heart rate was irregularly irregular, tachycardic without rubs, murmurs, or gallops. She had diffuse inspiratory and expiratory wheezing, with tachypnea and mild respiratory distress. She had no lower extremity edema, erythema, or redness. Labs were significant for an elevated troponin I 0.24 (normal value ≤0.05 ng/mL) and B-natriuretic peptide 4,776 (normal value 0-900 pg/mL). Her complete blood count, coagulation studies, and remainder of her labs were unremarkable. 

**Figure 2:**
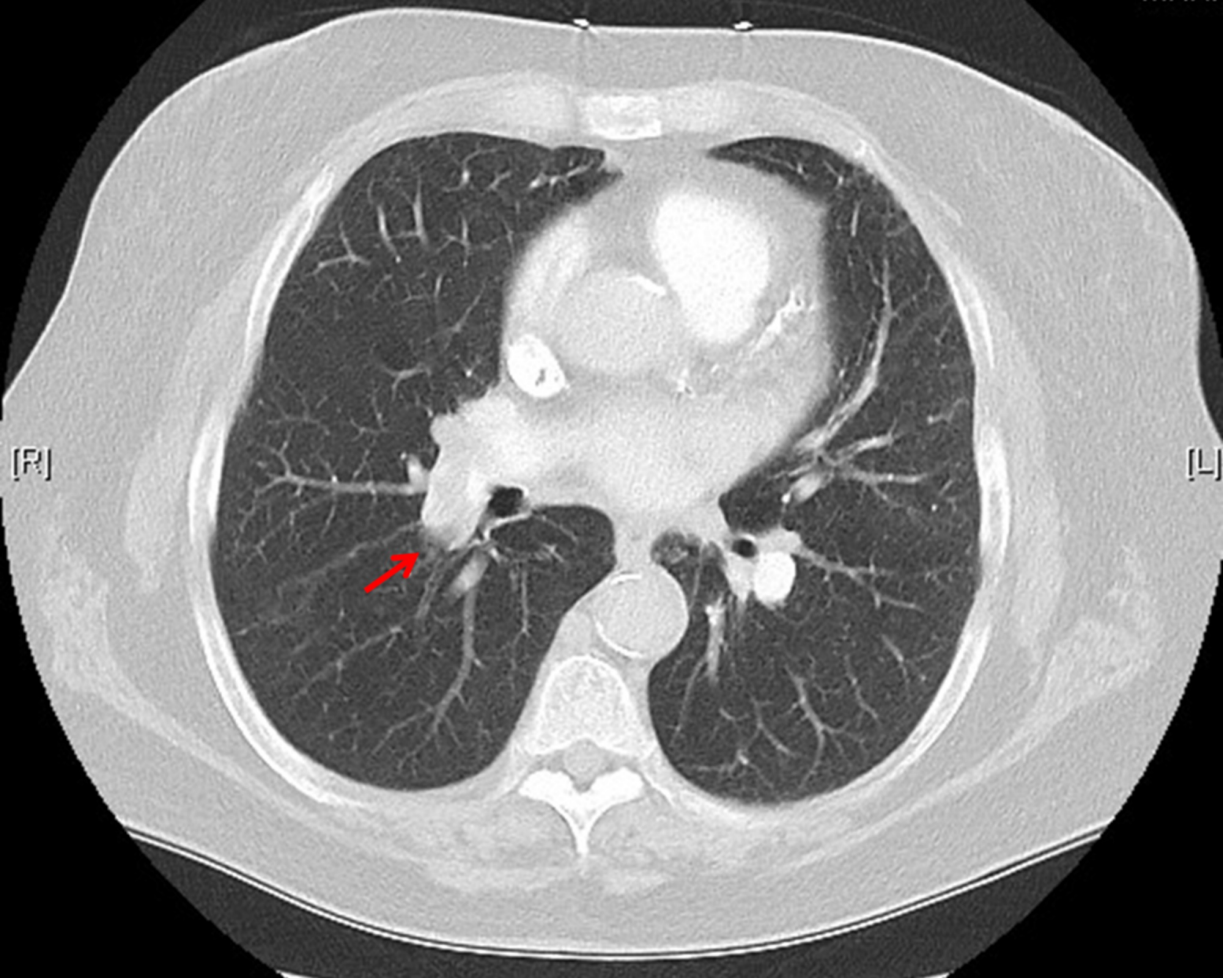
CT angiography of the chest with right main pulmonary embolism (red arrow) and upper and lower segmental emboli.

Given her degree of hypoxia and known findings of DVT, a CT angiography of the chest was ordered and demonstrated right main, upper and lower lobe segmental pulmonary emboli (Figure 2). Heparin bolus and drip were initiated, and interventional radiology was consulted for possible EkoSonic endovascular system (EKOS, catheter-assisted thrombolysis) treatment of PE. The decision was made to pursue EKOS, and the patient was admitted to the ICU. She underwent COVID testing on her second day of admission, and it was positive. The patient remained in the hospital for several days and was discharged on apixaban without any further complications.

Case 2

A 55-year-old male with past medical history significant for hypertension, asthma, and hypothyroidism presented to the emergency room complaining of fever, cough, chills, abdominal pain, and diarrhea. The patient was seen in the emergency room three days previously for the same complaint and discharged home with oseltamivir despite a negative influenza rapid test. The patient returned to the emergency room for worsening symptoms and was re-evaluated. His re-evaluation, including a chest X-ray, did not reveal any abnormalities. He was discharged home again. The patient returned a third time two days later with persistent fevers, chills, abdominal pain, vomiting, diarrhea, and now shortness of breath.

His initial vital signs were blood pressure 93/64 mmHg, temperature 38.4°C, heart rate 102 beats per minute, respiratory rate 19 breaths per minute, and oxygen saturation 99% on room air. Upon examination, the patient was awake, alert, oriented, and appeared ill. Lung sounds were clear and equal bilaterally with no signs of respiratory distress. Skin was pink, warm, and dry. He had moist mucosal membranes. His abdomen was tender in the left lower quadrant (LLQ) without rebound or guarding. The patient had labs ordered, a chest X-ray, CT of the abdomen/pelvis, blood cultures, COVID testing, and repeat point-of-care influenza test.

**Figure 3:**
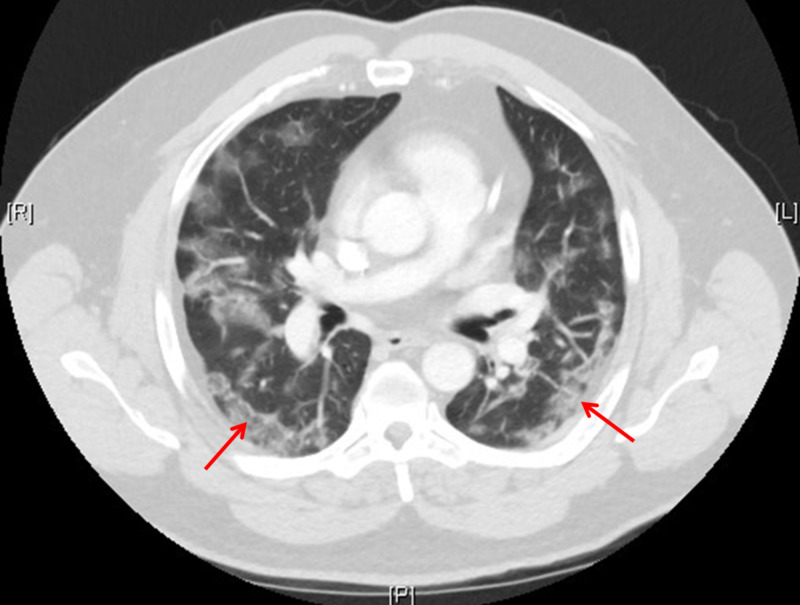
CT of the chest with bilateral ground-glass opacities (red arrows).

His labs were significant for an elevated creatinine of 1.50 mg/dL (normal <1.12), an aspartate transaminase (AST) of 240 units/L (normal 10-37), an alanine aminotransferase (ALT) of 282 units/L (normal 12-78), and a procalcitonin of 0.34 ng/mL (normal <0.05). Complete blood count showed platelets 109,000 platelets/μL (normal 150,000-400,000). His chest X-ray now demonstrated bilateral peripheral consolidations, highly suspicious for a viral infectious process. The CT of the abdomen/pelvis was normal except for patchy bilateral predominantly peripheral ground-glass airspace opacities within the lung bases, concerning for atypical pneumonia or viral pneumonia (Figure 3). Other etiologies, such as acute respiratory distress syndrome (ARDS), were also considered.

The patient was given antibiotics as part of the sepsis bundle as he met sepsis criteria as he was tachycardic, febrile, and had respiratory and GI symptoms. He was admitted and during his hospital course, his respiratory status continued to decline despite noninvasive oxygen therapy. His COVID test returned positive. He was started on a heated high flow nasal cannula, and he remained in respiratory distress with hypoxemia with a PaO_2_ of 62%. The patient was subsequently intubated and taken to the ICU.

While in the ICU he was noted to experience recurrent thrombosis of his central and arterial lines, requiring multiple replacements despite prophylactic anticoagulation (5,000 units subcutaneous heparin every eight hours). The patient further developed limb ischemia to his bilateral lower extremities and right arm. The patient underwent hypercoagulable workup, which demonstrated a low level of protein C activity and IgG and IgM phospholipid antibody elevation. The patient eventually succumbed to his illness.

Case 3

A 53-year-old man with past medical history significant for hypertension and prior smoking presented to the emergency department (ED) with complaints of fever, dyspnea, and cough that had started four days prior to presentation. He reported the fever had been progressively worsening since onset with a max temperature of 103°F at home. He also reported myalgias, fatigue, and decreased appetite. He mentioned that one of his coworkers had tested positive for COVID-19 after having similar symptoms.

His triage vitals were significant for a temperature of 37.6°C, a respiratory rate of 24 breaths per minute, a heart rate of 118 beats per minute, a blood pressure of 135/87 mmHg, and an oxygen saturation of 79% on room air. On physical examination, the patient was awake, alert, oriented, and in no distress. Lung sounds were clear and equal bilaterally with no signs of respiratory distress. There was no increased work of breathing. The patient spoke in full sentences; however, he did have to pause during longer sentences. His skin was pink, warm, and dry with moist mucosal membranes. His abdomen was soft and nontender.

Labs and a chest x-ray were ordered in addition to blood cultures and COVID-19 testing. His labs were significant for leukocytosis 13.6 x 10^3^ cells/mm^3^, D-dimer 1.30 mg/L (normal <0.49), hyponatremia 134 mEq/L (normal 135-145), lactic acid 2.4 mmol/L (normal <1), AST 86 units/L, lactate dehydrogenase (LDH) 746 units/L (100-190), and procalcitonin 0.43 ng/mL (<0.05). His chest x-ray demonstrated a mild perihilar interstitial infiltrate or edema pattern. 

During his clinical course in the ED, the patient had improved oxygenation on 6 L nasal cannula to 92% saturation. Empiric antibiotics and intravenous fluids were started for suspected sepsis. The patient was admitted to the hospital for acute hypoxemic respiratory failure requiring supplemental oxygen. On day 2 of hospitalization, the ICU team was consulted by the hospitalist and infectious disease for evaluation for increased respiratory effort. The follow-up chest x-ray also revealed increasing alveolar infiltrate, and the patient’s COVID-19 test was positive. On evaluation by the ICU, the patient endorsed worsening difficulty breathing, particularly with movement despite receiving 15 L via nonrebreather (NRB) mask. He was tachycardic to 105 beats per minute, had an oxygen saturation of 90% on 15 L NRB mask, and labored respirations at a rate of 23/min. His breath sounds were diminished bilaterally. 

**Figure 4:**
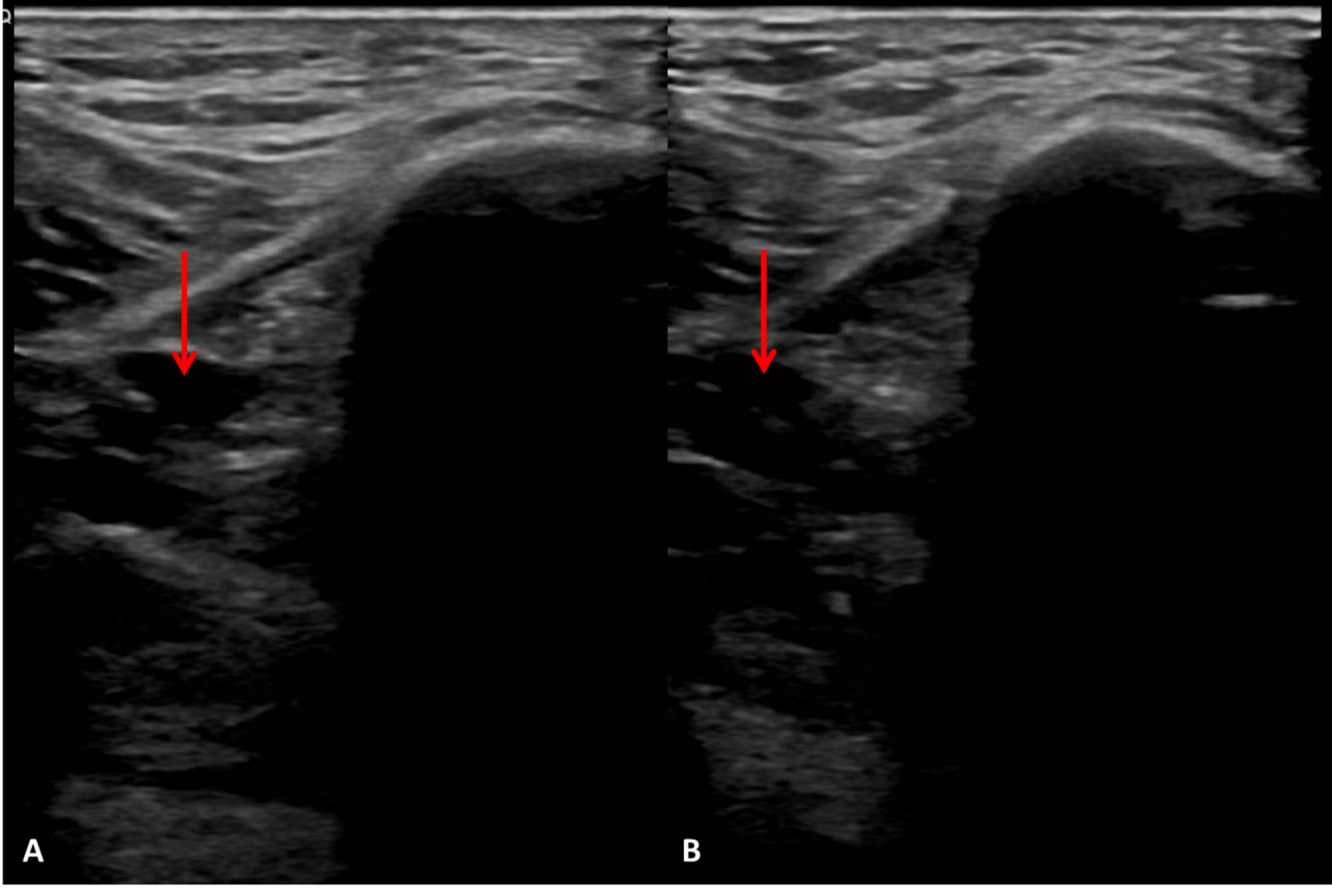
Ultrasonography of the left posterior tibial vein (red arrow) without compression (A) and with compression (B).

The decision was made to transfer the patient to the ICU for observation. His respiratory distress progressed, and the patient was intubated and sedated for mechanical ventilation. The patient’s urine output declined since admission to the ICU with concern for acute tubular necrosis possibly secondary to a thrombotic event. On day 4 of hospitalization, the decision was made to empirically start heparin drip (titrated to an activated partial thromboplastin time value 1.5 to 2.5 times the mean normal value) after the patient remained anuric. On day 9 of hospitalization, the patient was found to have a left lower extremity DVT in the posterior tibial vein despite empiric therapeutic level heparinization (Figure 4). He was eventually deemed a poor candidate for long-term anticoagulation for bleeding risk and had an inferior vena cava (IVC) filter deployed. The patient was discharged on hospital day 36 to a long-term acute care hospital after remaining afebrile for three consecutive days.

## Discussion

While the presenting symptoms of COVID-19 have been primarily respiratory in nature, these cases from our hospital demonstrate the sequela of the hypercoagulable state observed in more critically ill COVID-19 patients. Laboratory values of many of the first COVID-19 patients, especially ICU-bound COVID-19 patients, in Wuhan, China included elevated D-dimer, prolonged prothrombin time, and activated partial thromboplastin time, and decreased fibrinogen levels, although another study demonstrated only elevated D-dimer but no changes in prothrombin time and activated partial thromboplastin time [3,4]. Our cases contribute to the clinical manifestations of those initially observed laboratory abnormalities. 

The extent of prothrombotic events was widespread with a variable response to prophylactic and therapeutic anticoagulation. Case 1 had few other risk factors for developing a DVT with progression to a submassive PE requiring anticoagulation and directed EKOS therapy with eventual transition to apixaban. Both cases 2 and 3 had more widespread thrombotic symptoms including clotting off central venous catheter and arterial access, acute kidney injury, and limb ischemia, all the while being on prophylactic followed by therapeutic heparin.

The hypercoagulable state of some of our COVID-19 cases was seen to progress systemically given multiple organ systems affected. A progression has been reported elsewhere from systemic thromboembolic events to disseminated intravascular coagulation and begs for additional investigation for appropriate anticoagulation given two of our cases remained on therapeutic heparin the entire time they formed the thrombi [5]. The complement system might play a role in the hypercoagulable state in COVID-19 which could suggest targeted therapies [6]. Another report describes a hypercoagulable state in COVID-19 related to the presence of antiphospholipid antibodies which could suggest a need for antiphospholipid syndrome related therapies like warfarin [7]. While the correlation between critically ill COVID-19 patients and the hypercoagulable clinical manifestations in those patients may be a function of the severity of their illness, the morbidity and mortality observed in our single center and elsewhere provides urgency to find appropriate therapies for these most vulnerable COVID-19 patients. 

## Conclusions

With targeted treatments for the respiratory symptoms of COVID-19 still being actively investigated, our cases suggest the need for investigation into appropriate anticoagulation treatment with the hypercoagulable clinical manifestations of COVID-19. Some but not all critically ill COVID-19 patients responded to therapeutic unfractionated heparin which warrants need for comparison of the effectiveness of other widely used anticoagulants, including low molecular weight heparins, warfarin, or any of the many direct-acting oral anticoagulants (DOACs). Our cases also demonstrate the absence of data to support which modality of prophylactic anticoagulation should be employed to prevent the progression of clinically significant thrombotic/embolic events (DVTs, PEs, limb ischemia, etc.) in COVID-19 patients.
